# Exploring layers of vulnerability during COVID-19: qualitative research with communities in Indonesia, Nepal, and Vietnam

**DOI:** 10.1186/s12910-026-01400-y

**Published:** 2026-03-02

**Authors:** Thao Phuong Tran, Manish Duwal, Diana Timoria, Ida Ayu Sutrisni, Yen Thi Hong Nguyen, Claus Bogh, Phong Thanh Nguyen, Aria Kekalih, Dewi Friska, Abhilasha Karkey, Raph L. Hamers, Sonia Lewycka, Mary Chambers, OUCRU Covid-19 Research Group, Choisy Marc, Day Jeremy, Dong Huu Khanh Trinh, Dong Thi Hoai Tam, Donovan Joseph, Du Hong Duc, Dung Vu Tien Viet, Fisher Jaom, Geskus Ronald, Ho Quang Chanh, Ho Thi Bich Hai, Ho Van Hien, Hung Vu Bao, Huong Dang Thao, Huynh le Anh Huy, Huynh Ngan Ha, Huynh Trung Trieu, Huynh Xuan Yen, Kestelyn Evelyne, Kesteman Thomas, Lam Anh Nguyet, Lawson Katrina, Leigh Jones, Le Kim Thanh, Le Dinh Van Khoa, Le Thanh Hoang Nhat, Le Van Tan, Lam Minh Yen, Le Nguyen Truc Nhu, Le Thi Hoang Lan, Nam Vinh Nguyen, Ngo Thi Hoa, Nguyen Bao Tran, Nguyen Duc Manh, Nguyen Hoang Yen, Nguyen Le Thao My, Nguyen Minh Nguyet, Nguyen Thanh Ha, Nguyen Than Ha Quyen, Nguyen Thanh Ngoc, Nguyen Thanh Thuy Nhien, Nguyen Thi Han Ny, Nguyen Thi Hong Thuong, Nguyen Thi Huyen Trang, Nguyen Thi Kim Ngoc, Nguyen Thi Kim Tuyen, Nguyen Thi Ngoc Diep, Nguyen Thi Phuong Dung, Nguyen Thi Tam, Nguyen Thi Thu Hong, Nguyen Thu Trang, Nguyen Thuy Thuong Thuong, Nguyen Van Vinh Chau, Nguyen Xuan Truong, Nhung Doan Phuong, Ninh Thi Thanh Van, Ong Phuc Thinh, Pham Ngoc Thanh, Phan Nguyen Quoc Khanh, Phung Le Kim Yen, Phung Khanh Lam, Phung Tran Huy Nhat, Rabaa Maia, Rahman Motiur, Thuong Nguyen Thi Huyen, Thwaites Guy, Thwaites Louise, Tran Dong Thai Han, Tran Kim Van Anh, Tran Minh Hien, Tran Tan Thanh, Tran Thi Bich Ngoc, Tran Thi Hang, Tran Tinh Hien, Trinh Son Tung, van Doorn H. Rogier, Vidaillac Celine Pascale, Vu Thi Ngoc Bich, Vu Thi Ty Hang, Yacoub Sophie, Van Vinh Chau Nguyen, Thanh Dung Nguyen, Manh Hung Le, Thi Loan Huynh, Thanh Truong Nguyen, Nguyen Huy Man Dinh, Van Hao Nguyen, Bich Thuy Duong, My Ngoc Nghiem, Phu Huong Lan Nguyen, Thi Ngoc Thoa Pham, Nguyen Phuong Thao Tran, Thi Lan Phuong Tran, Thi Tam Uyen Le, Thi Thanh Tam Tran, Thi Ton That Bui, Kim Nhung Huynh, Tan Tai Ngo, Nguyen Hoang Tu Tran, Trong Vuong Vo, Thi Bich Ty Dinh, Thi Dung Le, Lam Uyen Thai, Thi My Tien Nguyen, Thi Thu Thao Ho, Ngoc Thao Nguyen, Ngoc Thien Vuong Huynh, Trung Trieu Huynh, Ngoc Phuong Thao Pham, Minh Phuong Phan, Soraya Weldina Ragil Dien, Livia Nathania Kurniawan, Mutia Rahardjani, Ralalicia Limato, Diana Timori, Fahmi Ramadhan, Summita Udas, Samita Rijal, Amit Gautum, Aakriti Pandey, Pratibha Thapa, Niharika Kharel, Manish Duwal, Dinesh Deokota, Rabi Shakya, Pawan Sharma, Anup Rajbhandari, Nguyen Thanh Truong, Bui Thi Hong Ngoc, Mai Thi Phuoc Loan, Jennifer Ilo Van Nuil

**Affiliations:** 1 Oxford University Clinical Research Unit, Hanoi, Vietnam; 2 Oxford University Clinical Research Unit, Kathmandu, Nepal; 3 Oxford University Clinical Research Unit Indonesia, Faculty of Medicine Universitas Indonesia, Jakarta, Indonesia; 4 Sumba Foundation, Sumba, Indonesia; 5 Oxford University Clinical Research Unit, 764 Vo Van Kiet, Quan 5, T.P, Ho Chi Minh City, Vietnam; 6 Hospital for Tropical Diseases, Ho Chi Minh City, Vietnam; 7 Department of Community Medicine, Faculty of Medicine, Universitas Indonesia, Jakarta, Indonesia; 8 Centre for Tropical Medicine and Global Health, Nuffield Department of Medicine, University of Oxford, Oxford, UK

**Keywords:** Vulnerability, Vulnerability assessments, Infectious diseases, Pandemic, Ethics

## Abstract

**Background:**

What does it mean to be vulnerable in a pandemic? COVID-19 and its complex ramifications have challenged policymakers and researchers worldwide to redefine and reassess vulnerability. This paper presents experiences of being vulnerable during the pandemic among communities in Indonesia, Nepal and Vietnam.

**Methods:**

From November 2020 to April 2021, we conducted qualitative research with communities in 13 locations including Jakarta, Bandung and Sumba in Indonesia; Morang & Sunsari, Kathmandu, Bhaktapur, Sindulpalchowk, Lower Mustang and Kapilvastu in Nepal; and Hanoi, Ho Chi Minh City, Nam Dinh and Dak Lak in Vietnam. We held discussions with key informants from local communities and healthcare systems to explore local ideas of vulnerability. Based on these discussions and findings from our media monitoring at all study locations, community members at high risk of adverse impacts from the pandemic were identified. Participants were purposively sampled to represent a range of age groups, occupations and levels of exposure to COVID-19 in the community and enrolled in semi-structured in-depth interviews about their experiences with the pandemic and public health responses. We analyzed and framed the results using Luna’s metaphor of “vulnerability layers”.

**Results:**

In total we held discussions with 16 key informants from local communities and healthcare systems and collected 93 in-depth interviews with community participants. Using the “vulnerability layers” metaphor, we describe how stimulus conditions, namely stringent public health responses to COVID-19, may aggravate vulnerabilities a person already experiences or create new vulnerability experiences (for example, stigmatized status). A vulnerable experience can be exacerbated by the layering of multiple vulnerabilities or their cascade effects; on the other hand, it can be mediated by personal or collective protective factors. Our findings also demonstrate the role of context, including local disease control approaches and sociocultural attitudes and norms, in both the activation of and protection against vulnerability.

**Conclusions:**

Vulnerability assessments should take into account potential stimulus conditions (including public health responses) and local complexities rather than relying on groupings of impacted people. An understanding of the local context is also essential to improve the uptake and effectiveness of public health measures.

**Supplementary Information:**

The online version contains supplementary material available at 10.1186/s12910-026-01400-y.

## Background

Globally coronavirus disease 2019 (COVID-19), caused by severe acute respiratory syndrome coronavirus 2 (SARS-CoV-2), has resulted in over 778 million reported cases and almost 7.1 million deaths as of April 13, 2025 [1]. At the start of the COVID-19 pandemic in the first quarter of 2020, there was academic and public health speculation about vulnerabilities: which groups or countries might be more vulnerable, for example, spaces in conflict or humanitarian crisis zones [2], those living in poverty or with inequalities [3], communities of healthcare workers [4], countries lacking health infrastructures [5], among others. These messages cautioning governments and public health officials to try to identify those more vulnerable were seen alongside media messages that virtually everyone could be vulnerable to COVID-19. The idea of vulnerability being completely inclusive was even relayed to our study team from an Indonesian key informant when asked who would be more vulnerable during the first year of the pandemic within their setting: “[*In COVID-19 times*], we are all vulnerable.” Of course, biologically, everyone was (and still is) vulnerable to acquiring COVID-19 but the individual and structural realities in different contexts impacted the level and depth of one’s vulnerabilities and/or their risk of moving into vulnerable spaces. While vulnerabilities related to COVID-19 and the public health response was at the forefront of our own research objectives, the concept rapidly became blurry as we realized the many complexities surrounding it.

‘Vulnerable groups’ are concepts that have been used for decades and across many disciplines. For example, in public health and population-based health, there are multitudes of surveys and scales to assess vulnerability as a way to define and classify those who may need more resources or targeted prevention programs related to health and health risk, (e.g. see FHI360 review covering a multitude of theories and assessments [6]). While well-intentioned, these wide-scale definitions and assessments typically fail in two main ways: first, they eliminate or discount the agency of those deemed ‘vulnerable’ and second, they remove aspects of local contexts and situations that can increase or diminish vulnerabilities or the risk of it [7]. These definitions tend to categorize and ‘other’ people and/or groups, which can worsen the vulnerabilities over time and reinforce the categories that may not have been appropriate when first assigned, what Ian Hacking terms as the ‘looping effect’ [8].

However, there have also been vulnerability assessment tools where local definitions and context specific realities are integrated into the assessments, typically stemming from the social sciences [9]. While there is not an agreed upon definition of vulnerability across the social sciences, there are several key features. For example, in anthropology, concepts surrounding vulnerabilities have been used for over 40 years in the context of climate change and disaster research where “vulnerability involves learning to become aware of disturbance, a practice that affects how we organize social worlds and our affective investments in them” but also learning to live with it [10]. Sociologists frame ideas of vulnerability as a multi-dimensional, ever progressing concept inherent in all social systems therefore at any given moment, individuals or groups are always more or less vulnerable [11]. Additionally, scholars argue that vulnerability is deeply linked to numerous forms of risk, poverty, and wider inequalities [12]. An elaboration of the concept related to health is research surrounding structural vulnerabilities, e.g. Paul Farmer’s concept of structural violence [13]. In a move away from focusing on individual risk and risky behaviors in initial HIV prevention work in early 1990 s, the concept centers on the myriad structures that could result (eventually) in health disparities [14] or the structures that enable risk [15]. In addition to the disruptions that vulnerabilities (or moments of it) bring to human life, these moments (or lifetimes) of vulnerabilities also have the potential to create new social support structures and often shape the nature other structures [16].

In the context of climate change, for example, the Intergovernmental Panel on Climate Change (IPCC) defines vulnerability as “the propensity or predisposition to be adversely affected and encompasses a variety of concepts and elements, including sensitivity or susceptibility to harm and lack of capacity to cope and adapt” with risk as a function of climate hazard, vulnerability and exposure [17]. Similarly, from the bioethics field, Luna [18] describes the concept of vulnerability using a layers metaphor where there may be multiple layers operating in different ways and as situations change vulnerabilities may also change (i.e. return or disappear). These layers and the way one experiences them are always dependent on the context. In this sense, the ways in which the layers interact is not universal, therefore different individuals experiences vulnerable spaces in different ways. Additionally, layers may cascade, that is enhance each other, while some layers may remain dormant unless a stimulus activates it, there is a large emphasis on how these layers function, not on categorization or stereotypes [18].

In this paper, we explore the experiences of community members in locations representing a range of urban and rural contexts across Indonesia, Nepal, and Vietnam related to the many forms of vulnerabilities and protective factors that they experienced. We present various layers of vulnerability by using case studies that show the overlapping vulnerabilities and how the context impacted how individuals experienced (or not) vulnerabilities during early COVID-19 times.

## Methods

### Setting

The data for this analysis are drawn from the first phase of the Social Science and Public Engagement Action Research (SPEAR) study, which is a mixed methods project including in-depth interviews, surveys, media monitoring, and digital diaries. The full study protocol was published previously, however, we describe relevant components for this manuscript here [19]. The objective of SPEAR was to explore the experiences and impacts of the COVID-19 pandemic and the public health responses for ‘vulnerable’ communities.

We collected data for six months from November 2020 until April 2021 from 13 locations across Indonesia, Vietnam, and Nepal with Jakarta, Bandung and Sumba in Indonesia; Morang & Sunsari, Kathmandu, Bhaktapur, Sindulpalchowk, Lower Mustang, Kapilvastu, in Nepal; and Hanoi, Ho Chi Minh City, and Nam Dinh, Dak Lak in Vietnam. We selected these locations because we already had existing relationships in place as well as embedded research staff on the ground and a long-standing program of research within each location. We found it essential to work where we already had established local teams in place to not only monitor the realities on the ground but also to understand the nuisances in the data. As we were in the middle of a pandemic, it was crucial to work with locations where ethics and local permissions could be set up more easily and with reliance on these relationships and teams.

We also selected these countries because they provided a diverse range of contexts, cultures, public health strategies and measures, and case numbers for us to explore and contrast people’s experiences of vulnerability. For example, during the data collection period, the COVID-19 situation in each country was evolving quite differently, including case numbers and the public health responses. Indonesia had a relatively steady number of cases during the data collection period; case numbers were going down in Nepal when data collection started but then started rising drastically at the end of the data collection period; and in Vietnam, there were very few cases reported during this period [20]. Researchers have developed a stringency index for the context of COVID-19, which is a measure made up of nine indicators (e.g. school and workplace closures, travel bans) ranging from 0 to 100, with 100 indicating the strictest levels. On the Our World in Data website, one can assess, at the level of a country, the stringency during specific time periods [21]. In Nepal, for example, the stringency increased simultaneously with the case numbers, while Indonesia’s index remained relatively stable, and even though Vietnam did not have many cases, the index was still in line with countries experiencing many cases [21]. While these countries adopted similar measures, they did so to varying extents. For example, Vietnam had a zero-COVID policy, Indonesia allowed isolation at home, and Nepal had long periods of lockdown.

### Recruitment and data collection

When we first designed the SPEAR study, we wanted to explore how the pandemic and/or the public health responses enhanced longstanding vulnerabilities or created new vulnerabilities. We did not want to include predefined groups that we assumed would be more vulnerable, instead we relied on key informants and media monitoring reports from each country to gain insight. When we initially developed the protocol, we aimed to recruit 1–2 key informants per site equaling 13–26 total informants across all sites. Key informants included people in hospital and community leadership positions linked to our research sites, and government officials. We used convenience sampling to recruit the key informants into the study, based on our existing networks and relationships.

Prior to community participant recruitment, we spoke with key informants and members of our research team regarding who, in their opinion, would be considered (more) vulnerable in the specific context of the pandemic at that time in the locations. Based on the media monitoring reports, key informants, and our research team, we created a list of groups who would potentially be more vulnerable. The inclusion criteria then included groups based on occupation (e.g. hospitality, retail, farmers), groups who had been quarantined and/or infected with COVID-19, among others. We aimed to purposively recruit 10–12 participants from each study site, with an estimated aim of 130–156 total participants across all sites, based on these groups but we also selected participants based on gender, age, and occupation to ensure a range of experiences. Exclusion criteria included those who would not provide consent and those under the age of 18.

At each site, social scientists collected the interview data either in person or remotely, based on the local situation/regulations at the time. We used a semi-structured interview guide that was developed by the study team with topics related to the project aims, as well as topics that became relevant as the pandemic situations changed. At the start of the interview, we included an open-ended question for the participant to recount their COVID-19 experiences at that time. Following this question, we had specific open-ended questions and probed as relevant. The SPEAR team from all locations had weekly debrief meetings in which we discussed the interview data, new questions/topics that might be relevant to add to the interview guide, and the general situation across the sites. We collected the interviews using the primary languages spoken in each context, audio recorded the interview (with explicit consent), we transcribed the interviews into language spoken, and translated all interviews into English so that all team members could review data from other sites. All participants provided written or recorded verbal informed consent prior to participating in the study. All individuals named in this manuscript are pseudonyms.

### Analysis

We analyzed the in-depth interviews and key informant discussions using a mixture of open and closed coding techniques [22]. Initially for the first round of coding, a team of 1–2 researchers per country coded the full data set with a closed codebook that was developed by the investigators based on literature and topics from the debrief meetings, a form of structural coding [22]. Many of the initial codes were broad so that the sub-codes could be inductively developed based on the context. The sub-codes and any new codes were then discussed at the debrief meetings with the full analysis team. We merged the overlapping sub-codes across locations, however, some of the codes only applied in particular locations.

In the second round of coding, which was conducted specifically for this particular analysis, one researcher from each country used the ‘relevant’ text from the coding [23] that focused primarily on two broad codes from the main coding: public health responses and impact of the responses, as a starting point. We then examined the excerpts of data from the full dataset to document how vulnerabilities worked across the sites, using the conceptualization of vulnerability from Luna [18], including documenting both stimulus conditions and protective factors, as well as acknowledging the layers aspects.

We discussed the findings as case studies across the sites, as well as reviewed the full set of transcripts for a more complete narrative from the participants. The second level and subsequent coding was done at the country level and then discussed together with the wider researcher team. Finally, we compiled the cases across the sites. The cases were selected to demonstrate the core concepts (i.e. stimulus conditions, layer interactions and cascading, protective factors) as well as reflect different contexts across the countries. They were not intended to construct complete categories of vulnerability, stimulus conditions, or protective factors. These cases are presented below.

## Results

We organized the findings into three main sections. First, we describe the demographics of the key informants and interview participants. Next, we describe vulnerabilities using Luna’s layers metaphor and demonstrate the ways in which the layers interact. Finally, we discuss protective factors as part of this conceptualization of vulnerability. In both sections, we analyze the salience of the particulars of context and provide examples from different settings.

### Description of key informants and interviewees

We recruited a total of 16 key informants across the sites including eight from Indonesia, six from Nepal, and two from Vietnam. In some settings, for example, in Vietnam, it was difficult to recruit key informants due to the nature of their jobs, for example, hospital officials and community leaders who were actively involved in the COVID-19 response. Overall, three informants were working in the government sector, eight were from leadership within the health system, and five were local leaders from the community (Table 1).

**Table 1:** Demographic characteristics of key informants, by country

	**Indonesia** ***n =* 8 (%)**	**Nepal** ***n =* 6 (%)**	**Vietnam** ***n =* 2 (%)**	**Total** ***N =* 16 (%)**
Sector				
Government	3 (37.5)	0 (0.0)	0 (0.0)	3 (18.8)
Hospital leadership	2 (25.0)	4 (67.7)	2 (100.0)	8 (50.0)
Community leadership	3 (37.5)	3 (33.3)	0 (0.0)	5 (31.2)

Overall, we interviewed a total of 93 participants from the community groups across the three countries from November 2020 to April 2021. Over half (52.7%) of the participants were female, 59.1% from urban sites, from a range of age groups, and were working in a variety of professions including teachers, people who stayed at home, business people, farmers, office workers, among other occupations (Table 2).

**Table 2:** Demographic characteristics of community interview participants, by country

	**Indonesia** ***n =* 30 (%)**	**Nepal** ***n =* 30 (%)**	**Vietnam** ***n =* 33 (%)**	**Total** ***N =* 93 (%)**
Gender				
Male	9 (30.0)	15 (50.0)	14 (42.4)	38 (40.9)
Female	21 (70.0)	10 (33.3)	18 (54.6)	49 (52.7)
N/A	0 (0.0)	5 (16.7)	1 (3.0)	6 (6.4)
Site				
Urban	20 (66.7)	18 (60.0)	17 (51.5)	55 (59.1)
Rural	10 (33.3)	12 (40.0)	16 (48.5)	38 (40.9)
Age group				
20–29	5 (16.7)	4 (13.3)	4 (12.1)	13 (14.0)
30–39	10 (33.3)	8 (26.7)	8 (24.2)	26 (28.0)
40–49	9 (30.0)	3 (10.0)	9 (27.3)	21 (22.6)
≥ 50	5 (16.7)	11 (36.7)	6 (18.2)	22 (23.6)
Not stated	1 (3.3)	4 (13.3)	6 (18.2)	11 (11.8)
Profession				
Farmer	3 (10.0)	4 (13.4)	3 (9.1)	10 (10.8)
Online motorcycle driver	1 (3.3)	0 (0.0)	1 (3.0)	2 (2.2)
Teacher	8 (26.7)	3 (10.0)	4 (12.1)	15 (16.1)
Student	2 (6.7)	0 (0.0)	1 (3.0)	3 (3.2)
Stay at home	10 (33.3)	1 (3.3)	1 (3.0)	12 (12.9)
Manual labor	1 (3.3)	0 (0.0)	2 (6.1)	3 (3.2)
Civil servant/Office worker/NGO	2 (6.7)	7 (23.3)	1 (3.0)	10 (10.8)
Retired	2 (6.7)	0 (0.0)	2 (6.1)	4 (4.3)
Business/Retail	0 (0.0)	8 (26.7)	5 (15.2)	13 (13.9)
Transportation	0 (0.0)	1 (3.3)	4 (12.1)	5 (5.4)
Hospitality	0 (0.0)	1 (3.3)	3 (9.1)	4 (4.3)
Other	1 (3.3)	5 (16.7)	6 (18.2)	12 (12.9)

### Vulnerabilities as “layers”

Initially, we relied on the key informant discussions for some guidance on whom to recruit as “vulnerable” participants. Multiple groups were identified by the key informants in all three countries, which indicated two main perceived types of vulnerability. The first type was vulnerability to COVID-19 itself. Listed groups under this type included people with comorbidities or compromised immune systems, and those with higher exposure such as family members of infected people, healthcare workers, laboratory technicians and contact tracing staff, especially those without adequate protective equipment. Meanwhile, groups such as wage workers, financially disadvantaged people, marginalized people, and those working in the travel industry referred to vulnerabilities to various economic impacts from the pandemic, in other words, those in precarious labor roles. Overall, there was no major difference among the countries in terms of the identified groups or how vulnerability was interpreted.

However, our analysis of community stories and experiences across various sociocultural settings indicated that COVID-19 as a whole-of-society phenomenon gave rise to more wide-ranging forms of vulnerability with complex dynamics. Rather than groupings of people with a “vulnerable” characteristic, vulnerability might be better conceptualized using the layers metaphor. Multiple vulnerabilities can layer on top of each other in an individual in their unique circumstances. For example, Sheela, a middle-aged Nepali woman, lived with different health vulnerabilities including thyroid, diabetes, heart and kidney diseases, and required regular hospital visits and expensive medications. This was accompanied by a layer of economic vulnerability since her sole source of income was her vending cart, which failed to earn enough income for the household during the pandemic and due to social restrictions imposed by the government. Finally, there was a mental health vulnerability as Sheela had previously been abandoned by her son and was then struggling to provide for her daughter-in-law and grandchild. The result was intense financial and emotional stress that complicated other aspects of her life.

**Fig. 1:**
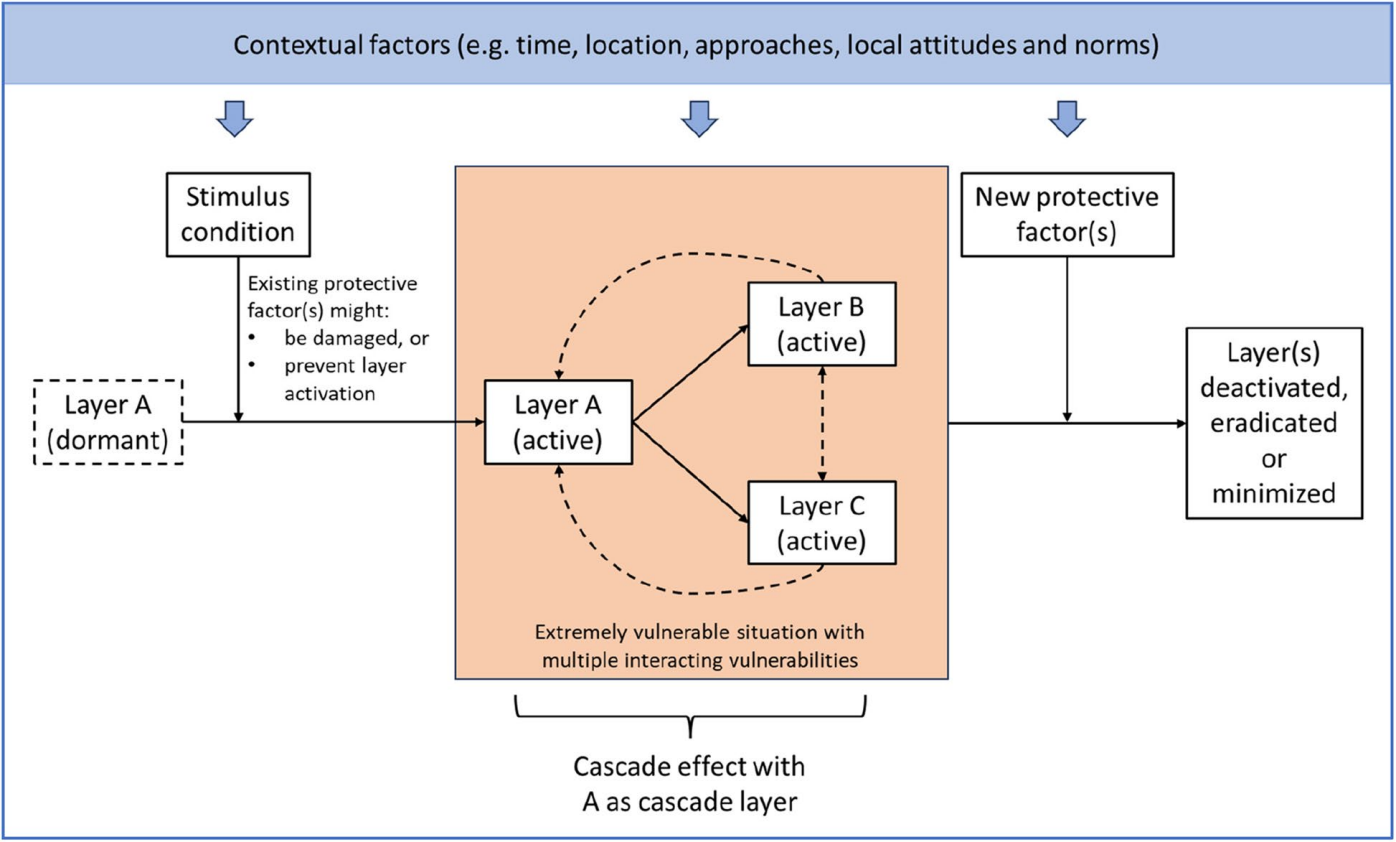
Visual representation of concepts

This layering of vulnerabilities is characterized by two key features, originally described by Luna and illustrated below in more detail. See Table 3 for a descriptive summary of the results and Fig. 1 for a visual representation.

**Table 3:** Summary of main findings

**Components (based on 12)**	**Key points**	**Examples**
Layer metaphor, as a concept	Multiple or different vulnerabilities can layer on top of each other in unique ways The idea of layers implies that vulnerabilities have a dispositional nature; they can be dormant and cause no immediate impact under normal circumstances	• During the pandemic, individuals might possess simultaneous vulnerabilities such as financial challenges, co-morbidities, mental health issues, illness related stigmatization, and impacts on social relationships • Local key informants identified certain groups who were vulnerable to infection or economic impacts. However, participants outside these groups were still found to experience extreme emerging vulnerabilities without recognition and support (see May’s case)
Cascade layers	In certain contexts, vulnerability layers may interact and influence one another. A cascade vulnerability layer may result in the exacerbation of existing layers or emergence of new ones and create a harmful impact. This is also called the cascade effect	• An economic vulnerability may lead to difficulties seeking care for co-morbidities (physical health vulnerability), and stress and anxiety (mental health vulnerability) • A vulnerability to COVID-19 related stigmatization may prevent an individual from accessing jobs, housing, and social opportunities, creating further related vulnerabilities
Stimulus conditions	A stimulus condition is an event or action that can cause dormant layers to be stimulated or other layers triggered In practice, the layers need to be identified and characterized through assessing stimulus conditions within the context	Public health responses (i.e. policies) often served as stimulus conditions in our research, such as: • Prolonged and large-scale lockdowns and mobility restrictions, with impacts on livelihoods and access to essential services; • Heavy-handed approaches to isolate people at risk of infection and disinfect their dwellings, which arouse fear, panic and stigmatization Certain contextual factors contributed to the likelihood and degree of vulnerability, including: • Negative attitudes toward and exclusion of infected people; • Local values and perceptions of individual responsibilities; • Expectations to follow traditional rituals and social etiquette
Protective factors	Protective factors might prevent or modify the damaging effects of potential vulnerability layers Protective factors might also be weakened or made inaccessible by stimulus conditions. Effective protective factors often employed local traditions, values, and community networks	• Personal protective factors: personal networks, knowledge and skills, practical and mental support from family and friends, etc • Collective protective factors: relief programs, protective policy actions, support and communication through local religious groups and community healthcare actors, etc

#### Layer interactions and cascade effects

While it is possible to identify separate categories of vulnerability, such as those of biological, socioeconomic and cultural origins, it might be more important to consider their interconnectedness. Vulnerability layers frequently interact and influence one another, resulting in the exacerbation of existing layers or emergence of new ones. In the previously described case of Sheela, various vulnerabilities overlapped and intensified one another. For example, her economic vulnerability was compounded by her health vulnerability, which required expensive medical care and medications. This, in turn, increased her financial stress and made it difficult to afford rent, food and other necessities.

*With no business, we were worried about the food. What if all gets finished up? (Sheela, aged over 50, cart vendor, Nepali)*

Additionally, her health vulnerabilities were exacerbated by the limited access to healthcare, medication and transportation during the pandemic, ultimately leading to her hospitalization. At the hospital, healthcare workers were hesitant to provide immediate treatment because she had symptoms suggestive of COVID-19 and required that she take a test. During this delay, her health continued to deteriorate.

*There was also no oxygen in the house. There was no bus. There was no transport. So I was taken to the hospital in an army van. That time they checked me for COVID-19, or else they would not have admitted me. (Sheela, aged over 50, cart vendor, Nepali)*

Furthermore, her son's abandonment and the family's financial struggles weighed down on her mental health. This made it more challenging for her to manage her physical health conditions, creating a positive feedback loop of vulnerabilities.

Beyond existing vulnerabilities, it is possible for new layers to arise due to a cascade effect. For example, May from Vietnam did not initially appear to be a “vulnerable” person. She was young, in normal health, and had a stable job at a local restaurant. However, the situation changed when she was infected with COVID-19, subjected to a contact tracing process and isolated at a hospital. Due to the fear and heavy stigmatization of COVID-19 patients in Vietnam at the time, she immediately became vulnerable to negative attitudes and discriminatory treatment.

*Except for my family, most people turned their back on me. After I returned from my [quarantine] leave, I lost my job. It took quite some time to find a new job. As for my apartment, the landlady kicked me out when I was in the hospital. She said that it had affected her very much, and that I had to move my stuff out of her place; she didn’t want me to stay there. But actually, I come from [another province]; I don’t have anyone here. Then she said if I didn’t have anyone here, she would just move my belongings out. It would be my responsibility if they were lost. (May, aged 20–29, restaurant worker, Vietnamese)*

Having lost her source of income and shelter, May acquired an unanticipated economic vulnerability, which was prolonged by the general economic situation: fewer job opportunities were available due to the pandemic, especially in the restaurant industry, which would have to restrict or cease operations whenever required by the government.

Another consequence of the stigma vulnerability layer was increased susceptibility to mental health problems. May was not only burdened with guilt because her infection had damaged the reputation of the restaurant where she had been working, but also traumatized by the constant blame and critical messages from her co-workers.

*Oh God, I felt so tired. They were always complaining and talking to each other on that [restaurant staff] group chat. A part of me felt really sorry for them, but I also thought that people were pretty selfish for not thinking about how I would feel reading those messages. I was influenced by them so I was quite upset…like I had thought they were nice to me but it turned out that I didn’t know them at all. (May, aged 20–29, restaurant worker, Vietnamese)*

This mental health vulnerability would endure even when she had managed to find a new job and new apartment. The trauma of stigmatization left her on edge and afraid to reveal her prior COVID-19 patient status to her new employer, co-workers and landlord.

*Even now, I still don’t have the confidence to tell them, because you never know… People are treating me well now, but what if things go back to how I was treated before? It would be like digging myself a hole; I would again feel terrible. (May, aged 20–29, restaurant worker, Vietnamese)*

May’s vulnerability to stigma acted as a cascade layer, triggering a chain reaction of economic and mental health vulnerabilities that did not previously exist. In the absence of a countermeasure, the cascade effect led to deeper and wider consequences, which she was neither prepared nor assisted to address. The case studies from Indonesia also showed the same vulnerabilities dynamics and therefore are not presented here.

#### Stimulus conditions: Impacts of policy and cultural norms

As layers, vulnerabilities may be dormant (i.e. not actively causing harm) until triggered by a certain event in a specific context. For Sheela, her health conditions were normally managed with weekly hospital visits and medications. When the government imposed a lockdown to control the pandemic, however, it became difficult to maintain the visits. Her health began to deteriorate and eventually required urgent medical attention.

*We did not go for the follow-up visit for 3 months. At that time I was not on dialysis. I had regular follow-ups for my kidney problem. During that period, fluid accumulated in the body. Then I started feeling sick and serious, and then my daughter-in-law told the police outside and the police van took me to the hospital during lockdown. (Sheela, aged over 50, cart vendor, Nepali)*

The lockdown in Nepal served as a stimulus condition, turning a manageable susceptibility into actual harmful consequences. Across all of our study sites, lockdowns and mobility restrictions triggered and increased vulnerabilities in various ways, including but not limited to livelihood impacts, difficulties accessing food, essential tools and health services, and mental health issues. The ensuing level of harm was influenced by contextual factors such as the duration and stringency of the control measures at each site. Before and during our study period, the government of Nepal imposed a total of 8 months of strict and partial lockdowns (March-July 2020 and April-September 2021). The prolonged closure and restricted operations of business led to a reduction in economic activity, resulting in job losses and decreased income for individuals and households. There were multiple reports of missed or delayed healthcare in general, which by contrast were rarely the case for participants in Vietnam, where lockdowns up until the time of data collection lasted no more than a month and were limited to certain outbreak areas. Mental health vulnerabilities were particularly likely to arise on top of other layers. For example, the uncertainty, fear, and social isolation associated with lockdowns gave rise to stress, anxiety, feelings of loneliness, depression, and purposelessness. The likelihood of domestic violence and family conflicts also increased. Over time, an initial economic vulnerability could evolve to severely threaten a person’s physical and mental well-being:

*There were thoughts that crossed my mind: one was that we might die, and another was that it would be better to commit suicide than to live without food. I pondered over these thoughts. Such thoughts used to arise and play in my mind. Thoughts kept playing in my mind like "how to do it [suicide]" and "what to do" used to come to me when I was alone. Even if I tried not to think about it, I couldn't help but think about it. (Rita, aged 40–49, beautician, Nepali)*

Evidently, longer lockdowns caused more vulnerabilities to be activated and were more challenging to address. In these situations, support policies such as relief or mental healthcare were often found to be unavailable or inadequate.

*The lockdown was imposed for a really long period of time and it would have been better had they [organized a relief program] later. They immediately did it after 15–20 days of lockdown. But when it [the lockdown] got extended to 6–7 months, they didn’t do anything [at that time]. (Suman, aged 30–39, shopkeeper, Nepali)*

Other public health responses such as quarantine and enforced isolation also frequently played a part in stimulating vulnerabilities. In Nepal and Vietnam, heavy-handed methods were employed to isolate suspected or confirmed patients from their community.

Security guards surrounded the place where the COVID positive person was found and then took that person with them; this is wrong. Instead of that, if the government had taken them to a normal environment for treatment then the citizens would not have been frightened. The government should not have sent an army of armed forces and surrounded the person with army rules. That is what the government did at first. They should not have done so. After these things, people got scared. (Hari, aged 30–39, municipality staff, Nepali)

They just come, round up entire families onto a vehicle and leave instantly. Then they sprinkle powdered lime, spray disinfectants, and make announcements, all of which cause people to panic and think of it like… I’m not old enough to have seen the plague or leprosy patients, but I feel like the fear in this case is comparable to the fear of leprosy patients. And nobody will dare to go past the area, even though life is still quiet. (Tung, aged 40–49, office manager, Vietnamese)

Fear was a common theme in these narratives which went beyond immediate experiences of psychological distress. Visible signs of danger – from armed forces to disinfectants, public loudspeaker announcements, and people being removed from their homes – engendered a widespread and enduring perception of COVID-19 infection as a threat, not only to one’s health but also to one’s normal life and community membership. As a result, local norms of exclusion concerning perceived risk carriers were created and reinforced. For the people who were infected in these instances, fear and avoidance continued after they recovered from the infection, creating vulnerabilities to stigmatization and exclusion from multiple services, opportunities and social groups, similar to May in our previous case study.

Cultural norms, including the emerging ones described above, contribute significantly to the likelihood and degree of vulnerability. Here by ‘culture’ we mean, following Napier [24], “…those contexts within which we make meaning, enact shared values and establish laws that guide what is ‘appropriate’ and ‘normal’” [24]. For example, data from Vietnam showed that it was appropriate and normal to prioritize collective safety over individual interests. Vietnamese participants were well-acquainted with the government’s appeals to stay at home, limit physical contact with others and report their travel history if they had COVID-19 related symptoms. Compliance was considered the norm and even a display of love for the country, according to a popular communication message. As a consequence, stigma experiences were often exacerbated on the grounds that someone had no awareness of the control measures or acted selfishly without any regard for the community. COVID-19 patients could face condemnation by people in the wider community, resulting in mental and physical trauma among other issues.

*Something that troubled me a lot mentally was the comments of the public. Some people reached me directly on the phone, some with encouraging words, but four to five people called to criticize and blame me. Some people sent me text messages accusing me of having no awareness and not quarantining even though I had been to [local outbreak cluster] and had experienced a cough and a fever. (Tung, aged 40–49, office manager, Vietnamese)*

Local ideas of ‘appropriate’ and ‘normal’ can be implicit yet widely influential, causing vulnerabilities to emerge in less obvious and unintended forms. In Sumba, Indonesia, Yuliana faced a difficult choice between health protocols imposed by the government and relationships with her community. During the pandemic, she had to hold a ‘grief ceremony’ 40 days after the passing of her father-in-law. Traditionally, this ceremony is attended by the whole family and their neighbors, and betel nuts are served to the guests. However, government restrictions at the time required people to keep safe distances and refrain from making physical contact at gatherings. Having the guests chew betel nuts and spit them onto the ground would go against the restrictions and increase the risks of COVID-19 transmission. On the other hand, holding the ceremony without serving betel nuts would imply a lack of respect for the guests and could cost the family their future chances to have such respect and support in return. To avert this potential vulnerability and protect her social standing, Yuliana decided to forgo the regulations.

*I think it can't be avoided. If there are lots of people like that, we always have betel nuts ready…because that's the truth. So, after all, we must serve betel nuts with coffee. (Yuliana, aged 30–39, farmer, Indonesian)*

The complexity and connection between these stimulus conditions indicate that vulnerability is not a permanent status, but rather vulnerable spaces deeply tied to the context that people may move in and out of, or manage to avoid entirely.

### Protective factors and vulnerability

While vulnerability layers can be shared by certain groups or people subjected to the same stimuli, their damaging effects might be modified in each case by protective factors. In our analysis, we identified multiple forms of protection and empowerment, ranging from personal networks, savings and competencies, to social support structures and policy responses.

#### Personal resources as protective factors

Despite the presence of a stimulus condition and relevant contextual factors, personal resources may enable a person to prevent vulnerability layers from being activated and cascade. In the following case study, Thanh was the chief executive officer of a company where he managed hundreds of employees. Similar to May in our previous example, Thanh tested positive for COVID-19 and was isolated for treatment at the same hospital. The events gave him a layer of vulnerability to stigmatization and potential damage to his business, which he fully understood from the outset.

*When I tested positive, I understood that I was no normal person. I’m the owner of a company with lots of partners. Some of them are banks with whom we have a million-dollar loan, vendors and suppliers who depend on us. So they needed to know the information from me, not rumors. You know back then, there were so many rumors around patients. And you know the personal information and travel itineraries that we gave to the Center for Disease Control were not well kept at all. They could easily be leaked and spread on different Zalo and Facebook groups. Those rumors would damage our reputation. Our business would be frozen because of me, and people would think that I had spread the virus to my staff. (Thanh, aged 40–49, business owner, Vietnamese)*

With this early realization, Thanh put his knowledge and skills to use. Having extensive experience as a social media user, he started a blog to publish information on his travel history, health conditions and daily life at the hospital. This ensured that his work partners, employees and other contacts received accurate information and retained their trust in the company. Furthermore, he compiled information from credible, research-backed sources to explain the illness to his readers, believing that fear and discrimination originate from a lack of understanding. As a result, he was able to prevent discriminatory attitudes and treatments during and after his hospitalization.

*When I came back, my neighbors celebrated with me. They even invited me to go on holiday with their family. Thanks to my communication, they knew that I was OK so they were not afraid of me, so no discrimination for me. (Thanh, aged 40–49, business owner, Vietnamese)*

His research and communication skills also proved useful in protesting against controversial policies by local authorities. After researching and discussing the strengths and limitations of diagnostic testing with medical doctors in his circle, he convinced the authorities to stop testing recovered COVID-19 patients every day for 30 days following their release from the hospital, thereby avoiding the unnecessary and potentially traumatic discomfort faced by many fellow patients.

In many cases, support from one’s family and friends also served to minimize vulnerability layers. When Ani and her husband got infected with COVID-19 and were isolated in separate places, a friend brought her essential supplies which she could not access from home.

*It was a friend of mine from another neighborhood that gave me help and supplies. So, she gave me some from... what's the name...’bansos’ [social aid] if I'm not mistaken. So, I heard that every local government has their own COVID-19 aid stockpile unless I'm mistaken. I heard that from the Village Consultative Council. So, every neighborhood has their own stockpile that they will distribute to their infected people. Meanwhile, I was given help from another neighborhood stockpile, not even my own neighborhood stockpile. (Ani, aged 30–39, housewife, Indonesian)*

An additional form of support was provided by her parents-in-law, who took over the daily care of her breastfeeding child while she was in isolation. This protected the child from infection and gave Ani peace of mind and positive energy because she had people she could trust.

#### Protection and empowerment through social support and policies

Where personal resources were constrained, such as in situations of restricted mobility, community-level protective factors often played a significant role. In Indonesia, local organizations and neighbors actively brought help to those in need. Each neighborhood, as we have seen in Ani’s narrative, had a social aid stockpile to distribute food and essential supplies to people who were infected and in-home isolation. Similar forms of aid were provided in Nepal by Tole reform committees (local level authorities) and members of the community. During this time in Vietnam, to strictly control community transmission, quarantine could take place at neighborhood rather than household level, and resources were directed by the local government to support the basic needs of those affected. These relief efforts absorbed some of the impacts of quarantine and mobility restrictions on people’s livelihoods and access to resources.

Local authority responses also provided protection against other types of vulnerability. For example, when people infected with COVID-19 faced stigmatization and threats of eviction from their own community in Nepal, the authorities responded by organizing neighborhood meetings. They ensured that people with COVID-19 were allowed to recover within their homes and had access to necessities.

*There was a pregnant woman in our ward. She had COVID-19, but her child did not. Some people intended to take her to the hospital. Later, other people in the community gathered and expressed their objection that she should not be staying in that area. They wanted her out of the area. We tried to convince them that forcing her to leave would pose even greater risks. She had the potential to transmit the infection to others as well. (Mala, aged 30–39, ward officer, Nepali)*

Another frequently mentioned protective factor was timely and accurate information related to COVID-19. In Sumba, Indonesia, where fake news was widespread on popular platforms such as Facebook and the television, healthcare workers drove around their village and provided updated information about COVID-19 with a speaker. This not only protected people in the village from false information but also instructed them on proper health protocols to stay safe.

*I have heard of the car with a speaker giving information about COVID-19. I think it was yesterday. We have heard of it. Yes, it was yesterday. I remember that we should keep our distance, and always wear masks, that's all we heard. (Yuliana, aged 30–39, farmer, Indonesian)*

#### Protective factors in context

Similar to vulnerability layers, protective factors are often influenced by the context. Stimulus conditions may strip people of protections normally available to them. For instance, income sources were one of the most severely impacted protective factors due to pandemic related restrictions, leading to financial vulnerabilities and ensuing problems, such as difficulties accessing necessities and domestic conflict.

In total I was in quarantine for a month and half, then when I came home, it had been two months without work. And with the pandemic, there were fewer jobs, so it was a headache for me. Meanwhile, I also had many things to worry about like rent, traveling costs and food while I was still jobless. And even when I found a job, the pay would be less than that of where I had been working for some time. (May, aged 20–29, restaurant worker, Vietnamese)

As soon as there is a shortage then some conflict starts in every home. If anything is abundant and prosperous then everything is good in that family; but as soon as it is insufficient, conflict arises here and there. (Anu, aged 30–39, teacher, Nepali)

Livelihood impacts were reported in a wide range of economic sectors and industries including tourism, transportation, hospitality, manufacturing, retail and agriculture, which meant that many people lost not only their main income but also backup sources. Practical or mental support from one’s family, an alternative form of protection, might also be hindered in certain situations. This was notably the case in Vietnam, where social distancing between households and community units was encouraged and often enforced to prevent any form of community transmission, despite the extent to which people traditionally rely on family and neighborhood ties in their daily life. For example, strict distancing between all households in one community forced a couple to take care of a newborn baby in isolation and without help from the child’s grandparents, who typically play an important role in childcare.

*Interviewer: Were there only you and your husband to take care of the kids? Or their grandparents…*

*Participant: Their grandparents didn’t dare to come because of the outbreak. There were many difficulties…we had to rely on ourselves.*

*Interviewer: So it was very hard to take care of the kids.*

*Participant: It was very hard during the outbreak, really terrible.*

*(H’Mari, aged 30–39, farmer, Vietnamese)*

On the other hand, contextual elements in the form of local traditions, values and religious networks were frequently leveraged to create and enhance protective factors. Among Nepalese communities, the practice of “namaste” was widely promoted during the pandemic to replace handshakes and hugs. This traditional greeting gesture involves placing the palms of both hands together and bowing the head, thereby reducing physical contact and the spread of the virus. To maintain social distancing guidelines, people were urged to perform Namaste from a safe distance of at least one meter rather than getting up close.

In Indonesia where approximately 87% of the population are Muslim [25], worship places became actively involved in COVID-19 related communication. They often provided accurate information and health recommendations, with religious leaders using their influence to encourage the adoption of health protocols. During supply shortages, mosques also joined primary healthcare centers to distribute free masks to the community.

Meanwhile, health literacy in Vietnam was promoted with appeals to put aside personal inconveniences and comply with the government’s measures for community safety. This approach relied on a culture of collectivist values, where social harmony is prioritized especially in times of crisis, and appeared to foster trust in the government and their recommendations. Similarly, these values substantially increased public support for other measures such as contact tracing and centralized isolation of infected people, which contributed to the successful management of outbreaks in Vietnam during the study period.

*In my opinion...contact tracing and other work done by the government as well as the Ministry of Health and health authorities haven’t really infringed upon people’s privacy… I think it’s all within a permissible extent. But each citizen should view things in a fair and positive manner and be cooperative, because this is not just for any individual. This is for the community’s good, so personally, I’m fully supportive. (Tung, aged 40–49, office manager, Vietnamese)*

While such measures were implemented at a cost, namely the previously described stigmatization of COVID-19 patients and privacy issues, they protected the majority of the community from infection and prevented further economic damage.

## Discussion

We explored layers of vulnerabilities in a range of communities in Indonesia, Nepal, and Vietnam, highlighting how some layers have the potential to cascade and enhance impact while others are dormant until stimulated by an event. The importance of protective factors and the particulars of the context are crucial for understanding vulnerabilities and creating appropriate responses. The examples from the case studies confirm the importance of the interplay between vulnerability layers, protective factors and stimulus conditions within specific contexts, and align with a complex conceptualization of vulnerability.

Our objective in this paper is not to list potential vulnerability layers, interactions and consequences in the pandemic, but rather to propose a change in how we conceptualize and assess vulnerabilities. At the start of the SPEAR study, we thought that some groups would be in more vulnerable spaces due to COVID-19 and the varied public health responses across our research sites. We therefore wanted to recruit from those who were more vulnerable, yet we did not know at that time who might fit into that category. We quickly discovered that this was not a straightforward task. Our attempt to map out vulnerabilities with key informants often ended up with a list of potential groups, not necessarily a description of potential layers and mediating factors. There are certain risks to this approach. Categorizing groups may have inadvertently missed those who were in a vulnerable space due to unassessed stimuli or the cascade effects of multiple layers. By applying Luna’s conceptualization [18] in our analysis, we found that vulnerability layers, apart from those of economic and biological origins, could also emerge in the form of stigmatization, psychological impacts, conflicts and tensions, and interact with existing layers. These findings resonate with previous studies looking into community experiences in the pandemic across different income settings and cultural contexts [26, 27]. While our key informants discussed stigmatization and mental health impacts in a separate part of the discussions focusing on healthcare worker experiences, they did not mention these issues when asked to identify forms of vulnerability. Based on our conversations with key informants, despite the demonstrated role of public health interventions such as lockdowns, mobility restrictions and quarantine/isolation in stimulating vulnerabilities, assessments of their unintended impacts appeared to be lacking across the study sites. For instance, while financial relief was mentioned as a countermeasure against economic impacts, there were indications that relief efforts did not match the scale of local restrictions or reach people in less apparent vulnerable situations. Many forms of vulnerability seemed to receive less attention, as we have seen in the case studies: for example, Sheela, as a chronically ill person, could not access regular healthcare or emergency treatment due to mobility restrictions and COVID-19 testing requirements. May continued to suffer on her own from the financial, social and psychological consequences of isolation and contact tracing. It was clear that forceful measures to control community transmission and strong communication messages reinforced negative attitudes towards the illness and discriminatory behavior. Therefore, affected individuals might feel discouraged from voicing their vulnerabilities and find themselves in situations of limited agency, where shocks from control measures may outweigh the impact of the disease itself [28]. Caution must also be taken on the other side of the extreme where everyone then could be classified as vulnerable thereby making the term useless [18]. An important note here is that the layers metaphor views vulnerability as a disposition rather than a permanent status. Identifying potential stimulus conditions, their probability of occurring, and estimated harm considering cascade vulnerability layers can provide a basis to rank the layers and determine priority actions [18].

The risks from rigid approaches to defining vulnerability indicate a need to continually perform and refine vulnerability assessments, which is in line with expert calls early in the pandemic to redefine vulnerability in the public health responses [29, 30]. We should use caution with assessments that do not take into account the complexities of local communities. Similar to this analysis, there have been definitions of vulnerability developed during COVID-19 that take into account the complexities and intersecting domains that make up vulnerabilities [28, 29]. More practically, A. David Napier created a manual called “The Rapid Assessment of Vulnerable Populations: A ‘barefoot’ manual” with the goal of assessing local vulnerabilities for resource allocation during ‘extreme social unrest’ [31]. According to this manual, as well as within our findings, specific contexts are crucial to understand in detail when conceptualizing vulnerabilities [31]. First, when we are trying to identify who may experience more distress during an outbreak or conflict situation, understanding the local context may help to determine potential stimuli that activate a dormant vulnerability layer. It could also provide ideas as to which layers might cause cascade effects that are particularly harmful and should be prioritized [18]. For example, a vulnerability assessment that relates heavy-handed contact tracing and quarantine approaches to negative sociocultural constructions of COVID-19 infections may draw attention to the wide-ranging consequences from stigmatization. Such assessments may benefit from a participatory approach to draw on local knowledge and structures, which has been found to improve applicability on a local level [32]. Further, results from a systematic review that focused utilizing the concepts of vulnerability within community engagement (in the context of infectious diseases) included only 15 studies but these studies all shared a common focus on history and structural factors, indicating the value of having a “vulnerability-informed approach in community engagement” [33].

In addition to demonstrating vulnerability layers, we attempted to map protective factors onto this concept. Protections, either in the form of personal or collective resources, could mean the difference between activating and averting a vulnerable space. This supports the proposed fluid conceptualization of vulnerability. Public health responses should aim not only to contain transmission but also to enhance these factors and build on the strengths, structures, and traditions of local communities. On the other hand, stimulus conditions can potentially neutralize or cut off access to protective factors. Stringent and restrictive responses to the pandemic should therefore avoid damaging the existing resources of individuals and communities that enable their resilience. Understanding the local context is important to not only maintain these resources but also improve the implementation and uptake of public health responses. For example, if health protocols prevent people from fulfilling their underlying social responsibilities, they may be forced to choose between the two, which could leave people in uncomfortable situations (e.g. foregoing the public health recommendations, as demonstrated in our study). Exploring and integrating local knowledge and social customs can enrich the ways in which public health interventions are conceptualized. Experience from previous epidemics suggests that interventions supported by community knowledge and action are likely to be more successful than standardized approaches [34]. However, Raymond and Ward found that these local knowledge systems were often only explored in a limited way or completely absent in qualitative and ethnographic research included in their review on community experiences of COVID-19 in low and middle income countries [26].

### Strengths and limitations

Working in multiple locations in three countries during the pandemic allowed us to collect a range of perspectives and experiences related to vulnerabilities, which was advantageous for conceptualizing the complexities and particularities of vulnerabilities. Recruitment of participants was also limited to the sites where we already had relationships and research teams embedded, which was good for maintaining trust with research communities, but this may have limited potential participants who could have different experiences in other sites where the context was quite varied. However, while the particular may be different, the conceptualization should remain the same. Finally, we collected data from November 2020 to April 2021, therefore the results only represent this timeframe which was before the Delta and Omicron periods of the pandemic. The pandemic responses, as well as people’s experiences and attitudes toward it likely changed over time as the pandemic progressed.

## Conclusions

In conclusion, in this manuscript, we explored a range of experiences that community members faced in several communities across Indonesia, Nepal, and Vietnam. We have demonstrated the complexities of the term ‘vulnerable’ and the importance of both protective factors and the contextual realities when thinking about how different people may be impacted differently during a pandemic and in response to pandemic measures. We noted how some vulnerability layers may cascade resulting in more intense impact; other layers become activated by external events. Overall, these examples provide an alternative pathway to exploring vulnerabilities and emphasize the importance of viewing vulnerabilities as a dynamic concept.

## Supplementary Information

Supplementary Material 1. In-depth interview guide for community participants

## Data Availability

The anonymized datasets analyzed during the current study are available from the corresponding author on reasonable request.

## Abbreviations

COVID-19: Coronavirus disease 2019
SARS-CoV-2: Severe acute respiratory syndrome coronavirus 2
SPEAR: Social Science and Public Engagement Action Research Project
