# Supplementary material for: Exploring layers of vulnerability during COVID-19: qualitative research with communities in Indonesia, Nepal, and Vietnam
Source: BMC Med Ethics. 2026 Mar 2;27:73. doi: 10.1186/s12910-026-01400-y (PMC13059250; doi:10.1186/s12910-026-01400-y)
Supplement: Supplementary file 1 — Supplementary Material 1. In-depth interview guide for community participants [file 12910_2026_1400_MOESM1_ESM.docx]

**Semi-structured interview – community**

In-person interviews will last between 1-2 hours; online interviews may be conducted over a series of shorter online interactions with the same participant. Questions might be re-phrased, when necessary, and additional topics and probes will be included, based on the responses of the interviewees, as well as information gathered during other data collection.

In general, all questions in this guide should be asked, however, the order can be edited to make the interview more conversational.

| **Ice breaker** |
| --- |
| **Informed consent**  If first interview, conduct full consent process; if subsequent interview, remind participant about nature of study and consent process. In all cases, ask participant if they have any questions about the study or their participation in this study. |
| **Participants’ information** |
| Participant ID  Age  Occupation  Gender  Have you been tested for COVID-19?  If yes, what was the results? |
| **Interview part 1: Open ended narrative of COVID-19 experiences** |
| [*Goal: to gain a narrative of experiences from the participant’s point of view prior to any focused questions to gain a picture of what their experiences include.]*   1. To start, we would like to know more about your experiences during the COVID-19 outbreak. After, we will ask more detailed questions but to begin, please tell us the story of your life during COVID-19. Please start the story where you like and take as much time as you need. 2. [*when they are finished*] Thank you for sharing your story. I would like to ask you more about [*insert 1-2 questions that you would like to probe on from their story*].   [*probe: during the narrative, try not to interrupt for details – note any questions you would like to probe on, use acknowledgement probes, e.g. ok, yes, mhhh and gentle probes, e.g. “is there anything else you would like to add” or “what else happened?” and then ask follow-up questions after.]* |
| **Interview part 2: How is COVID-19 understood in different locales?** |
| Topics: COVID-19 knowledge, health care seeking changes, information     1. Could you tell me more about what you know about COVID-19?    1. [*potential probes: where did it come from? How does it spread? Is it dangerous? Who gets it? Is anyone at more/less risk to acquire it? Add additional probes from survey results]* 2. What are the symptoms of COVID-19? 3. Before the COVID-19 outbreak, what would you do if you had similar symptoms to those you just listed? 4. Has that changed since COVID-19?    1. Why or why not?    2. *[if yes]* Could you elaborate on how it has changed? 5. Where do you and your family obtain information about COVID-19? [*probes, if necessary*: *TV, social media (Facebook, Zalo), radio, friends, family, healthcare professionals]* 6. Could you tell me more about the types of information provided from these sources? [*probe: What are some of the exact messages or information (‘facts’) that you remember from these sources?]* 7. Are there sources that are not trusted by the community in general? If yes, how do you know if the information is good or bad (or true or ‘fake’)? [*probe about any survey or media results]* |
| **Interview part 3: Disease transmission, adherence to public health guidelines, and ad hoc harm reduction** |
| We have a few questions about various measures you and your family have taken in response to COVID-19. By family, for these questions, we mean those who are living in your home with you.   1. Who all is living in your home right now? [*probe: # of people, generations*]    1. Has this changed since before COVID-19? If yes, how and why? 2. What are the three biggest changes that have occurred for you and your family since COVID-19 (or during COVID-19)?    1. [*probe for details on specific situations, for each challenge]* Did you have these challenges prior to COVID-19?    2. *[if had challenges before]* Has this challenge become better or worse for you and your family? Why and how? 3. What do you think you should you do to protect yourself and your family during this outbreak or future outbreaks? [*probe: for each method, ask their perception of the effectiveness and how easy or difficult it is for them and their family to do it and why*] 4. [if not discussed in q12] What was it (or is it) like **to isolate at home**? Was there anything that made it more difficult or easier for you and your family? If yes, what were they? [*probe: work related, care taking responsibilities outside the home, family interaction, family relationship dynamics, etc*]. 5. How did **the isolate at home** order change your practices for events that typically involve a social gathering? (*probe if necessary e.g. religious activities, weddings, funerals, births, graduation, other common rituals for that society*).    1. Could you give an example of how you changed your activities related to these events? 6. How did/does the COVID-19 and the public health response impact your daily life? [*probe about livelihoods, healthcare seeking (routine + vaccinations*)] 7. [*if active or past contact tracing in community*] What do you think about the contract tracing that governments are conducting in order to identify contacts of those with confirmed COVID-19?    1. Do you think this is an effective measure? Why or why not?    2. Do you think it is an invasion of personal privacy? 8. [*if active or past contact tracing in community*] Have you or a family member been involved in contact tracing: either had to provide information on your contacts or have been informed that you were potentially exposed through a contact?    1. If yes, what was that experience like? |
| **Interview part 4: Social stigma and ‘othering’ associated with COVID-19** |
| 1. Are there specific groups who are more responsible for the spread of COVID-19 in [*insert country*] or more broadly?    1. [If yes] Who are these groups?    2. [If yes] Why do you think they are more responsible for the spread of COVID-19? 2. Do you think *all* health care workers and health care staff should temporarily live in a space away from their families during COVID-19? Why or why not? 3. [*if not mentioned*] Do you think health care workers who are working at quarantine facilities or hospitals with known COVID-19 cases should temporarily live in a space away from their families? Why or why not? 4. [*add country specific questions, as appropriate*] |
| **Interview part 5: Wrap up** |
| 1. Is there anything else you would like to add to our discussion related to your experiences of COVID-19? |
